# Prevalence of Hypertension in Sudanese Patients With Gouty Arthritis

**DOI:** 10.7759/cureus.24248

**Published:** 2022-04-18

**Authors:** Sufian M Khaild, Amro M Fagir, Ziryab I Taha, Awadelkareem A Elshareef, Mohammed H Mohammed, Khalda M Saeed, Elnour M Elagib, Elwalied M Ibrahim, Jimmy William

**Affiliations:** 1 Internal Medicine, Nile Valley University, Atbara, SDN; 2 Internal Medicine, Al Ain Hospital, Abu Dhabi, ARE; 3 Rheumatology, Sudan Medical Specialization Board, Khartoum North, SDN; 4 Internal Medicine, University of Bahri, Khartoum, SDN; 5 Urology, Nile Valley University, Atbara, SDN; 6 Internal Medicine, Atbara Teaching Hospital, Atbara, SDN; 7 Biochemistry, Nile Valley University, Atbara, SDN; 8 Rheumatology, Omdurman Military Hospital, Omdurman, SDN; 9 Internal Medicine, International University of Africa, Khartoum, SDN; 10 Internal Medicine, Sligo University Hospital, Sligo, IRL

**Keywords:** arthralgia, allopurinol, uric acid, hypertension, gouty arthritis

## Abstract

Background

In this study, we aimed to study the frequency of hypertension in Sudanese patients with gouty arthritis attending the largest three tertiary hospitals in Khartoum and correlate it with serum uric acid levels.

Methodology

An observational, descriptive, cross-sectional, hospital-based study was conducted in rheumatology clinics in Khartoum state, Sudan, from August 2020 to January 2021 involving 100 participants. Data were collected, prepared, and analyzed using SPSS version 25.0 (IBM Corp., Armonk, NY, USA).

Results

In this study, 100 participants were enrolled. The majority were males (79%), with 45% of the participants in the age group of 61-75 years. Overall, 89% of participants had symptoms of gouty arthritis, with the knee being the most common joint affected in 27% of participants. Most participants had a uric acid level above the target (6 mg/dL). The most frequently used uric acid lowering agent was found to be allopurinol in 85% of the patients. Furthermore, among those with gouty arthritis, 51% had hypertension with nearly half being insufficiently controlled. The frequency of undiagnosed hypertension among the participants was found to be 19%, which was statistically significant among gouty arthritis patients (p-value < 0.0001). Upon further analysis of our hypertensive participants, 79.5% of males (n = 35) had high blood pressure levels, which was statistically significant as well (p-value = 0.005), with the highest prevalence being among the age group of 61-75 years. Of those who were hypertensive, 51% had a history of concomitant comorbidity. Overall, 90% of the hypertensive participants (n = 40) had joint symptoms. Moreover, serum uric acid level was above the target in 93% of the participants.

Conclusions

Hypertension was found to be the most frequently recognized comorbidity in gouty arthritic patients, with more than a third remaining undiagnosed. Moreover, the male gender was a significant risk factor for hypertension among the gouty arthritis participants. Nevertheless, most patients with high blood pressure levels had concurrent elevated uric acid levels.

## Introduction

Gout is a disorder characterized by the deposition of monosodium urate (MSU) crystals in the synovial fluid. Although hyperuricemia should precede the deposition of MSU crystals, differentiating between hyperuricemia and gout is essential as, in many cases, hyperuricemia may not result in crystal deposition. Gout has different stages, including hyperuricemia, deposition of MSU crystals, inflammatory response to MSU crystals, and tophi formation [1]. It can be diagnosed by either microscopic examination of synovial fluid for MSU or a scoring system comprising symptoms, signs, and laboratory findings [2].

The incidence and prevalence of gout are rapidly rising [3,4]. Gout may shortly exceed 3% of the adult population [1]. The aging of the general population may explain its increasing prevalence, associated comorbidities, sedentary lifestyle, or poor nutritional habits [5]. Gout is associated with a significant risk for cardiovascular disease, affecting mortality and morbidity rates (hypertension, stroke, obesity, and dyslipidemia) [4]. Additionally, it is recognized as a marker for the quality of life [6].

Many studies have addressed the comorbidities that are associated with gout [3,4]; for instance, in one study, the presence of monosodium crystals was associated with coronary artery and thoracic aorta calcification, which might be explained through MSU-induced inflammation in vascular tissue [7]. Gout was associated with an increased prevalence of hypertension, ischemic heart disease, diabetes mellitus, hyperlipidemia, and chronic kidney disease. The increased prevalence was proportionate with the duration of gout [3,8]. Furthermore, hypertension was the most common comorbidity associated with gout, and this association appears to be bidirectional [9].

Another related entity is reactive oxygen species (ROS) that may accumulate in hyperuricemic patients due to uric acid oxidation, leading to endothelial dysfunction and resulting in atherosclerosis, ischemic heart disease, insulin resistance, and metabolic syndrome [3]. On the other hand, hypertension can damage glomerular arterioles, causing glomerulosclerosis and, therefore, renal insufficiency and high uric acid [9]. Hyperuricemia, renal failure, diabetes mellitus, and metabolic syndrome can affect high blood pressure management as it becomes more refractory when associated with these comorbidities [6]. Therefore, our study aims to assess the prevalence of hypertension among gouty arthritis patients in correlation with serum uric acid.

## Materials and methods

Study design, area, and duration

This was a cross-sectional, descriptive, hospital-based study conducted in rheumatology clinics in Khartoum state, Sudan. These included rheumatology clinics in Ibrahim Malik Teaching Hospital, Omdurman Teaching Hospital, and Military Teaching Hospital. The study was conducted from August 2020 to January 2021.

Study population

We included all adult patients attending the rheumatology clinic who were diagnosed with gouty arthritis, whether having another rheumatological disease or not, and regardless of their medical history or associated comorbidities. In addition, we included previously known hypertensive patients and those newly diagnosed if confirmed.

We excluded patients with no clear diagnosis of gouty arthritis, those with other rheumatological diseases and/or other comorbidities with no confirmed diagnosis, as well as those not attending follow-up if newly diagnosed with hypertension.

Sampling

Adult patients attending the rheumatology clinics from August 2020 to January 2021 were included. The study participants were already diagnosed with gouty arthritis through clinical manifestations, serum uric acid, and/or joint aspiration by a consultant rheumatologist.

Sampling technique

Total coverage was chosen because only a few patients with gouty arthritis attended outpatient clinics (around one to two patients per day).

Data collection method and tools

We used questionnaires for data collection. Participants were interviewed by an investigator and questionnaires were filled out. Blood pressure was measured in both arms in a sitting position after 10 minutes of rest. It was measured three times, with the first measurement discarded (to avoid any possible effect of anxiety). Finally, an average value of the second and third measurements was taken for systolic and diastolic blood pressure. According to the National Health Institute for Health and Care Excellence (NICE) guidelines [10], the blood pressure of participants was considered elevated if above 140/90 mmHg among those aged below 80 years or above 150/90 among those aged above 80 years.

Patients with high blood pressure who had no previous history of hypertension were followed up further before labeling them as newly diagnosed with hypertension.

Finally, blood samples were obtained for serum uric level analysis. The serum uric acid target was below 6 mg/dL (normal range: adult male, 4.0-8.5 mg/dL; adult female, 2.7-7.3 mg/dL) according to the recommendations from the American College of Rheumatology (ACR) guidelines for the management of gout 2020 [11] and the 2018 updated European League Against Rheumatism (EULAR) evidence-based recommendation for the diagnosis of gout [12].

Data management and statistical analysis

SPSS version 25 (IBM Corp., Armonk, NY, USA) was utilized to analyze data. Age, gender, blood pressure, comorbidities, medications, uric acid levels, and gout duration were assessed.

Ethical consideration and approval

Ethical clearance was obtained from the Education Developmental Centre of Sudan Medical Specialization Board and related authorities in hospitals. Written informed consent was obtained from all participants. Participants had the right to no harm (privacy and confidentiality using a coded questionnaire). Additionally, participants had the right to benefit from researcher knowledge and skills. Finally, all possible precautions for the prevention of coronavirus disease 2019 (COVID-19) spread were taken, including washing hands, wearing face masks, and securing the distance between the researcher and patients.

## Results

This study involved 100 participants, of whom 79% were males and 21% were females. According to age clustering, most of the study participants (45%) were in the age group of 61-75 years, followed by 36% being in the 46-60-year age group. Our study demonstrated gout duration distribution among the study participants and revealed that 65% of participants were diagnosed with gout for less than three years and only 18% had gout for more than five years. Overall, 89% of the participants had symptoms of gouty arthritis during the interview and 59% had oligoarthritis (two to four joints involved). The most affected joint was the knee (27.6%), followed by the ankle (14.8%), wrist (13.3%), shoulders (11.2%), and fingers (10.9%).

The majority of the participants (90%) had a uric acid level above the target (>6 mg/dL). Allopurinol was the most used uric acid-lowering agent in 85% and only 2% used febuxostat. Hypertension incidence was 51% and was the most common comorbidity. The frequency of diabetes mellitus was 12%. Furthermore, 8% of the study participants had a history of both hypertension and diabetes mellitus. The frequency of rheumatoid arthritis was 9%. It was also noted that 19% of study participants were diagnosed with osteoarthritis besides gouty arthritis. Of our study population, 44% had uncontrolled blood pressure, and 43.2% (n = 19) were newly diagnosed, reflecting more than one-third (37%) of the hypertensive participants in our study (Table 1). In addition, about 96% of the hypertensive participants who were previously undiagnosed had significantly elevated uric acid levels, as shown in Table 2.

**Table 1:** Association between blood pressure levels among hypertensive patients and characteristics of the study group. *Comorbidities include diabetes, osteoarthritis, rheumatoid arthritis, ischemic heart disease, hyperlipidemia, and renal impairment.

P-value	Blood pressure		Parameter
	High	Normal	
0.005	Gender		
	79.5%	20.5%	Male
	20.5%	79.5%	Female
0.77	Age group		
	4.5%	14.3%	30–45
	27.3%	28.6%	46–60
	54.5%	42.9%	61–75
	13.6%	14.3%	75–90
0.73	Presence of comorbidities*		
	50%	57.1%	Yes
	50%	42.9%	No
0.22	Presence of diabetes mellitus		
	11.4%	28.6%	Yes
	88.6%	71.4%	No
0.84	Presence of osteoarthritis		
	25%	28.6%	Yes
	75%	71.4%	No
0.82	Presence of rheumatoid arthritis		
	11.4%	14.3%	Yes
	88.6%	83.7%	No
0.12	Presence of joint symptoms		
	90.9%	71.4%	Yes
	9.1%	28.6%	No
0.67	Uric acid level		
	93.2%	71.4%	Less than 6 mg/dL
	6.8%	28.6%	More than 6 mg/dL

**Table 2:** Association between diagnosed/undiagnosed hypertension and serum uric acid level.

P-value	Hypertension		Parameter
	Undiagnosed	Diagnosed	
0.04	Serum uric acid level		
	96.9	78.9	Above 6 mg/dL
	3.1	21.1	Below 6 mg/dL

Nevertheless, upon assessing the blood pressure levels among our hypertensive participants, we found that 86.3% (n = 44) had a level above the target, of whom 79.5% (n = 35) of these were males and 20.5% (n = 9) were females. More than half of the hypertensive participants who had high blood pressure levels (54.5%) were in the age group of 61-75 years. Overall, 51% of our hypertensive participants had a concomitant disease. An analysis of these concomitant diseases showed that osteoarthritis was the most common in 19% of participants, followed by diabetes mellitus and rheumatoid arthritis, being 12% each. Furthermore, in patients with gouty arthritis and concurrent hypertension, a correlation between joint symptoms and blood pressure levels was made which demonstrated that 90.5% (n = 40) of those with symptomatic arthralgia had high blood pressure levels simultaneously. In addition, in hypertensive patients with uncontrolled blood pressure, 90% had uncontrolled uric acid levels (more than 6 mg/dL) as well, as shown in Table 1.

**Figure 1:**
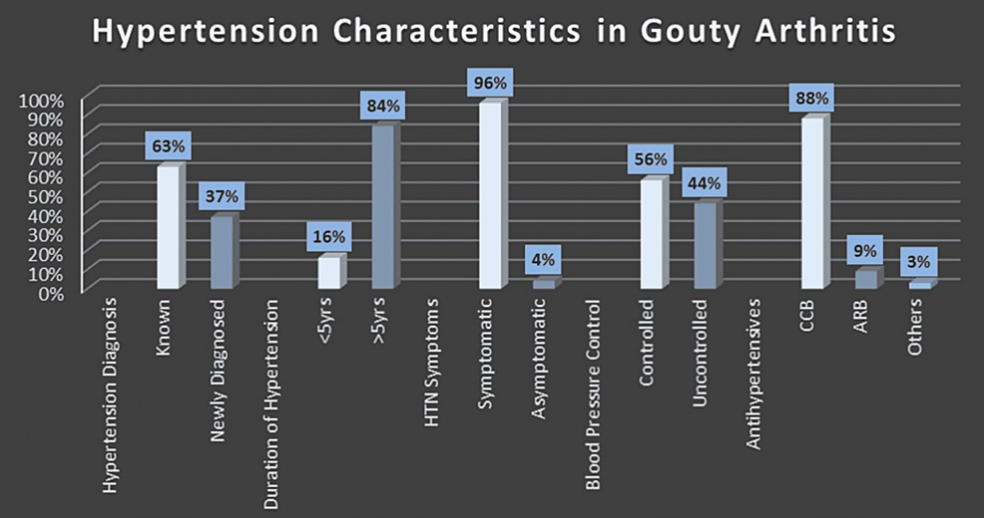
Hypertension characteristics in the gouty arthritis study group.

On further analysis of the participants who were known hypertensive, 84.4% had hypertension for more than five years. Almost all our hypertensive participants had no symptoms of hypertension during the interview. The most frequent antihypertensive medication class used by participants who were diagnosed with hypertension was calcium channel blocker, specifically amlodipine (88%), followed by angiotensin-converting enzyme inhibitors (ACEIs)/angiotensin II receptor blockers (ARBs) (9%) (Figure 1).

## Discussion

Gouty arthritis is a very common entity that presents early in life with a higher prevalence in males [1]. In this study, 79% of the participants were males, which is similar to the findings reported by Jeyaruban et al. and Mahmoud et al., with 82.9% and 81.2%, respectively [4,13]. The overall age distribution of the participants showed that 45% were between the ages of 61 and 75 years, followed by 36% between the ages of 46 and 60 years, which is comparable to the results reported by Singh et al., who concluded that the prevalence of gout increases proportionally with age [7]. Nevertheless, Mahmoud et al. demonstrated the highest prevalence in the age group of 46-60 years (46.5%) [13]. Furthermore, Jeyaruban et al. found that less than 1% were below the age of 30 years, almost similar to our study, where we had none below this age group [4].

The main involved joint in our symptomatic gouty arthritis patients was the knee (27.6%). This result corresponds with the review by Angelo et al., which stated that around 80% of initial flares involve the knee or first metatarsophalangeal joints [2].

On assessing the uric acid-lowering agents used by participants, 85% were using allopurinol, which is similar to findings by Mahmoud et al. (90%), and slightly higher than those reported by Jeyaruban et al. (76%) [4,13].

Interestingly, our study revealed that hypertension was the most frequent comorbidity (51%). This result is comparable to those reported by Jeyaruban et al. (60.8%) and Huang et al. (97.8%), with the latter reporting higher prevalence rates of hypertension [3]. Furthermore, on comparing our findings with those of Mahmoud et al., which were both conducted in Khartoum (Sudan) but seven years apart, we observed that the prevalence of hypertension among gouty arthritis patients is rapidly increasing from 18.8% to 51% [13]. This could be explained by the fact that most of our patients had elevated serum uric acid levels, which may raise the likelihood of gouty arthritis flares and increase the risk of developing hypertension, given that a significant correlation between high uric acid levels and undiagnosed hypertension has been demonstrated in this study. Another possible explanation is that the most frequent age group in our study was 60-75 years, while the most common age group in the study by Mahmoud et al. was 46-60 years, considering that increasing age is considered a risk factor for developing hypertension [14].

Diabetes mellitus was the third most frequent comorbidity (12%) in our study, three times higher compared to Mahmoud et al. (4%) [13]. In addition, less than one-third of our study population had osteoarthritis concurrently with gouty arthritis, which is not an uncommon finding as it has been described previously by Roddy et al., who presented a significant association between the acute attack of gout and the presence of clinically diagnosed osteoarthritis, especially at the first metatarsophalangeal joint, mid-foot, knee, and distal interphalangeal joint. This association can be explained by the changes that occur in the osteoarthritic joint, predisposing it to MSU crystal formation. Moreover, osteoarthritis may determine which joint will be affected by an acute attack of gouty arthritis [15].

Another concomitant entity with gouty arthritis described in our study is rheumatoid arthritis, which was found in 9% of patients. This was observed before by Chiou et al., who addressed the frequency of hyperuricemia and gout in patients with rheumatoid arthritis. Although the coexistence of both is rather infrequent, evidence of MSU deposition in joints was demonstrated in a proportion of rheumatoid arthritis patients in different studies [5,16]. Furthermore, it has been reported previously that patients with rheumatoid arthritis who are hyperuricemic have a greater comorbidity burden regarding hypertension and chronic kidney disease versus those who are normouricemic [15].

The most common antihypertensive medication used by the participants was calcium channel blocker (CCB), especially amlodipine (88%), followed by ACEIs/ARBs (9%), with losartan being used by 5% of the participants. This is in line with a recommendation by Bardin et al., who concluded that beta-blockers, ACEIs, non-losartan ARBs, thiazide, and loop diuretics are associated with an increased risk of gout [8]. Consequently, CCB and losartan are the preferable antihypertensive choices for gout owing to their uricosuric properties [4].

As mentioned above, 51% (n = 51) of the participants had high blood pressure, 37% (n = 19) of whom were undiagnosed. This is similar to results by Gragson et al. and Wang et al., who reported a significant increase in the relative risk of hypertension in hyperuricemic patients [17-19]. Male gender was a significant risk factor for hypertension in our gouty arthritis participants as 79.9% of the hypertensive participants with uncontrolled blood pressure levels were males (p-value < 0.005). More than half of our hypertensive participants had a concomitant disease other than hypertension, thus highlighting the role of the other comorbidities in developing hypertension and the importance of screening and managing these diseases, even though there was no significant association between these diseases and blood pressure levels in our study. Nevertheless, in hypertensive participants, an association between elevated blood pressure and high serum uric acid level was observed, which is comparable to the results by Cho et al. and Juraschek et al., who concluded that hyperuricemia increases the risk for uncontrolled blood pressure [20,21]. Additionally, Cho et al. reported that hypertensive patients with hyperuricemia could have uncontrolled blood pressure despite good adherence to antihypertensive medications [21].

This study has a few limitations, primarily being hospital-based with a small sample size. Moreover, the cross-sectional design does not permit determining the casual correlation between risk factors and outcomes. Another limitation is that many of the participants with gouty arthritis were diagnosed clinically and by uric acid level rather than identification of uric acid crystals in joint aspiration, as facilities and expertise to analyze joint fluid were not available. Several criteria have been developed previously to assess the diagnosis of gouty arthritis without aspiration [22-24]. In addition, all participants were diagnosed by rheumatology specialists. Despite these limitations, this study is novel as it reflects the prevalence of hypertension among gouty arthritis patients and the concurrent risk factors in Sudanese patients attending the largest three tertiary hospitals in Khartoum.

## Conclusions

Hypertension is the most frequent comorbidity in gouty arthritis patients, with the majority of participants who had elevated blood pressure levels concurrently demonstrating high uric acid levels and joint symptoms. Furthermore, the male gender was found to be a significant risk factor for hypertension in gouty arthritis participants.
